# Plasminogen activator inhibitor-2 and impaired fibrinolysis in pregnancy and sickle cell anemia

**DOI:** 10.1007/s00404-023-07121-6

**Published:** 2023-07-04

**Authors:** Durjoy Shome, Lamiaa Al-Jamea, Alexander Woodman, Abdel Halim Salem, Moiz Bakhiet, Safa Taha, Amarjit Kaur Sandhu, Fatimah S. Al-Yami, Khawaja Bilal Waheed, Elmoeiz Ali Elnagi, Mohammed Almish, Jenifer Vecina Quiambao

**Affiliations:** 1 Department of Pathology, Arabian Gulf University, Manama, Kingdom of Bahrain; 2 Department of Medical Laboratory, King Fahad Military Medical Complex, Dhahran, Kingdom of Saudi Arabia; 3 School of Health Sciences, University of Salford, Manchester, United Kingdom; 4 Deanship of College of Medicine & Medical Sciences, Arabian Gulf University, Manama, Kingdom of Bahrain; 5 Department of Molecular Medicine, Arabian Gulf University, Manama, Kingdom of Bahrain; 6 Department of Obstetrics and Gynecology, Salmaniya Medical Complex, Manama, Kingdom of Bahrain; 7 Department of Medical Laboratory, King Fahad Military Medical Complex, Dhahran, Kingdom of Saudi Arabia; 8 Radiology Department, King Fahad Military Medical Complex, Dhahran, Kingdom of Saudi Arabia; 9 Department of Clinical Laboratory Sciences, Prince Sultan Military College of Health Sciences, Dhahran, Kingdom of Saudi Arabia; 10 Vice Deanship of Postgraduate Studies and Research, Prince Sultan Military College of Health Sciences, Dhahran, Kingdom of Saudi Arabia

**Keywords:** Plasminogen activator inhibitor-2, Euglobulin clot lysis time, Gene polymorphism, Antigen levels, Sickle cell anemia, Pregnancy

## Abstract

**Purpose:**

This is the first study that aimed to determine antigen levels in plasma and genotypes of PAI-2 in pregnant and non-pregnant homozygous sickle cell anemia (SCA) patients.

**Methods:**

The study subjects were all Bahraini females in the reproductive age group. The study population included 31 pregnant homozygous SS (SCA) patients. Three control groups were also studied to evaluate the effect of pregnancy and SCA on PAI-2 levels and fibrinolysis: (1) 31 healthy non-pregnant volunteers; (2) 31 cases of normal pregnancy; and (3) 20 non-pregnant SCA patients. Pregnancies were screened in the second (TM2) and third (TM3) trimesters. Global coagulation, fibrinolysis rate (euglobulin clot lysis time, ECLT), PAI-2 antigen (ELISA), and PAI-2 Ser(413)/Cys polymorphism (restriction fragment length polymorphism analysis) were determined.

**Results:**

Feto-maternal complications were documented in both pregnancy groups. PAI-2 antigen levels were undetectable in the non-pregnant groups, but was quantifiable in both pregnant groups. Impaired fibrinolysis rate and rising PAI-2 levels with progression of pregnancy were observed in both healthy and SCA subjects. These changes were more prominent in SCA, although the rise in ECLT was less steep and PAI-2 antigen levels were not significantly different compared to normal pregnancy in the third trimester. No correlation was observed between PAI-2 genotypes and plasma antigen levels. Also, no significant difference in feto-maternal complications was found in normal (n = 25) versus SCA pregnant patients (n = 30).

**Conclusions:**

These observations suggest that with progression of pregnancy, increasing PAI-2 levels contribute to the hypercoagulable state, particularly in SCA patients.

## What does this study add to the clinical work

**Table:** 

This is the first study on antigen levels in plasma and genotypes of PAI-2 in pregnancy associated with homozygous sickle cell anemia (SCA). Significant coagulation system activation with fibrinolysis in a background of impaired fibrinolysis characterized pregnant SCA patients. No correlation was observed between PAI-2 genotypes and plasma antigen levels. This study highlights the possibility that high levels of PAI-2 and impaired fibrinolysis in pregnant SCA patients, particularly in the second trimester, is a potential therapeutic target for intervention in selected high-risk cases.	

## Introduction

Plasminogen activator inhibitor type-2 (PAI-2) is a member of the serine protease inhibitors (SERPIN) super-family. It exhibits inhibitory activity toward the plasminogen activators u-PA and t-PA [1, 2]. PAI-2 is mainly produced by trophoblasts in the placenta and may thus contribute to the inhibition of fibrinolysis which occurs during pregnancy [1–3]. It is also found in human white blood cells such as monocytes, macrophages, eosinophils, and cells of epithelial lineage such as keratinocytes and certain tumor cells [1]. The level of PAI-2 in circulation is too low (< 15 ng/ml) to be easily detectable. It increases in certain circumstances such as pregnancy and in association with several malignancies including childhood lymphoma and acute monocytic leukemia [3]. The gene encoding PAI-2 is located on chromosome 18q. It is 16.5 kb long and consists of eight exons and seven introns [4]. Human PAI-2 is a single chain protein of 415 amino acids. It exists predominantly in a non-glycosylated intracellular form. However, a relatively small percentage of PAI-2 is secreted as a glycosylated protein found in the plasma of pregnant women [4–7].

Two polymorphisms have been previously identified in the non-coding region of the gene [8, 9]. Other polymorphisms have been identified in the coding region of the gene. Based on these coding region polymorphisms, two variants of the PAI-2 gene have been described: variant A consisting of Asn, Asn, and Ser and variant B consisting of Asp, Lys, and Cys at positions 120, 404, and 413, respectively. It has been suggested that the additional cysteine in variant B contributes to the PAI-2 polymerization [3, 10, 11]. Buyru et al. (2003) showed a strong association between PAI-2 genotype AA and myocardial infarction risk [10]. Earlier research by Foy et al. (1997) showed no significant difference in genotype distribution between patients with coronary heart disease and a control group [3]. Mercado et al. (2007) examined the association between PAI-2 polymorphism and systemic lupus erythematosus with antiphospholipid syndrome in a Mexican family, but no significant association could be demonstrated [11].

In normal pregnancy, the balance of hemostasis changes toward hypercoagulability, which reduces hemostatic complications associated with childbirth [12]. In addition, most blood coagulation factors (FXIII, XII, X, VIII, VII, fibrinogen) and von Willebrand factor levels increase during pregnancy [12]. Factor XI is the only blood coagulation factor that may be decreased, maybe due to increased consumption. However, factor XI activation is the key step in stimulating thrombin formation. It is also possible that during normal pregnancy, factor XI levels are physiologically reduced to counterbalance the increase in other coagulation factors [12, 13]. While blood coagulation inhibitors are mainly unchanged, free and total S protein levels markedly decrease, and tissue factor pathway inhibitor (TFPI) levels increase. Thrombomodulin levels also increase during pregnancy [12].

Fibrinolytic capacity is diminished during pregnancy, mainly because endothelium-derived PAI-1 increases during the later stages of pregnancy, and placenta-derived PAI-2 is detectable in the plasma during the first trimester and increases substantially throughout pregnancy [13, 14]. In early pregnancy, t-PA activity is close to the standard range seen in non-pregnant women, and there is a gradual decrease with gestational age. This decrease in t-PA activity is due to increased t-PA inhibitors PAI-1 and PAI-2 levels [13, 15, 16].

The hypercoagulable state in sickle cell disease is well documented and is implicated in the increased risk of feto-maternal complications in pregnant SCD patients. Furthermore, the biochemical balance in normal pregnancy, as outlined above, is tilted in favor of coagulability. The rationale for conducting this study was twofold: (a) the fibrinolytic state in pregnant sickle cell patients has not been adequately studied and (b) PAI-2 levels and its possible genetic links have never been investigated in pregnant SCD patients previously. Therefore, this study aimed to determine antigen levels in plasma and genotypes of PAI-2 in pregnancy associated with homozygous SCA. To examine the separate effects of pregnancy and sickle cell disease on these parameters, the study included three control groups: (a) healthy non-pregnant, (b) healthy pregnant, and (c) non-pregnant SCA.

## Methods

### Study sample

The study included n = 113 Bahraini women in the reproductive age (16–46 years) who were examined at the Salmanyia Medical Center between January 2006 and December 2006. Patients were selected by participating investigators from the departments of hematology–oncology and obstetrics and gynecology. After the purpose of the study was explained to them, all subjects included in the study were asked to complete and sign an informed consent form prior to participation.

### Data collection

The test group included 31 cases of pregnancy with SCA. For comparison, 31 healthy non-pregnant females, 31 normal pregnancies, and 20 female non-pregnant SCA patients were included. Patients with SCA had not received hydroxyurea therapy, received no blood transfusion in the month prior to sample collection, and were in stable condition. The pregnant cases were recruited in second trimester (TM2) and were followed up in the third trimester (TM3) of pregnancy to compare the changes in test parameters in the two phases of pregnancy. The pregnancy cases were followed up in the department of obstetrics and gynecology during the course of pregnancy up to the time of delivery. At each antenatal visit, inquiries for blood transfusions and hospitalizations were made.

Blood samples were collected in vacutainers from each participant. One 2.5 ml sample was collected in ethylene diamine tetra acetic acid (EDTA) and used for complete blood count (CBC), DNA extraction, and high-performance liquid chromatography (HPLC). Two samples (2.5 ml each) were collected in tri-sodium citrate solution with theophylline, adenosine, and dipyridamole additives (CTAD anticoagulant, Becton Dickinson, U.K) and used for coagulation testing, PAI-2 antigen assay, and euglobulin clot lysis time (ECLT). The samples in CTAD were kept in ice following collection and processed within 2 h to minimize platelet activation. These tubes were cold-centrifuged (4 ℃ at 1200–1500 rpm for 15 min). Separated plasma from one tube was processed for coagulation testing and ECLT immediately. Plasma from the second tube was stored at – 80 ℃ and processed for PAI-2 antigen assay within 30 days. The EDTA sample was analyzed by the Coulter LH 750 automated blood cell counter. Hemoglobin fractions were identified and quantified by HPLC, using the BioRad Variant analyzer (Japan). Plasma PAI-2 levels were measured by ELISA technique using IMUBIND^®^ PAI-2 ELISA kit (American Diagnostica Inc., Stamford, CT, USA). ECLT was tested using the method described by Laffan and Manning (2001).

The PAI-2 gene polymorphism was determined using the RFLP technique. Primers were designed from the published cDNA sequence to amplify a 123 bp region containing the two amino substitutions at positions 404 and 413 and consisted of the following:

PAI-2 F: 5′-ACAGTT TGT GGC AGA TCA TCC-3′

PAI-2 R: 5′-AAA AAT CTT TTG CAG AAG CAGC-3′.

The relevant gene segment was PCR amplified using primers 5′ and 3′ of the SNP, and the amplicon was then subjected to digestion with a restriction enzyme. The restriction enzyme MwoI used in the procedure recognizes the nucleotide sequence:
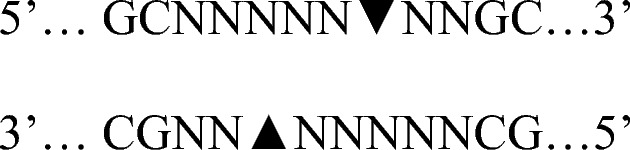


After restriction, the PCR product was electrophoresed on polyacrylamide or agarose gel, and the DNA fragmentation pattern was revealed by UV light. The procedure followed was similar to the method described by Foy and Grant (1997) [3]. The ECLT was measured manually by precipitating the citrated plasma with cold acetic acid. The resulting precipitate fraction (euglobulin) contained plasminogen activator, plasminogen, and fibrinogen. Most of the plasmin inhibitors remained in solution. The precipitate was redissolved, fibrinogen was clotting with thrombin, and the clot lysis time was measured. Detailed protocols are available upon request.

### Statistical analysis

Data were analyzed using SPSS software version 15. Student’s *t* test was used to test for differences among study groups related to hematological variables, including those related to CBC parameters, coagulation, and fibrinolysis studies. Mann–Whitney test was used for variables with non-normal distributions. Differences between levels of variables in the second and third trimesters were analyzed by paired samples *t* test.

One-way ANOVA was applied to test for significance of association between PAI-2 genotypes and antigen levels. Differences between observed genotype frequencies and expected frequencies (Hardy–Weinberg equilibrium) were assessed using an exact test using a Markov chain provided by the Arlequin software.

## Results

In total, 113 women of reproductive age from 16 to 46 years, with an average age of 32.1 ± 7.0 years, were enrolled in this study. The study population was divided into four groups: a test group of pregnant SCA patients (n = 31); healthy non-pregnant females (n = 31), normal pregnant females (n = 31), and non-pregnant females with SCA (n = 20).

The mean Hb value for healthy pregnant females in TM2 showed mild but significant reduction compared to the non-pregnant group (*P < *0.001). In TM3, the Hb level in this group did not show significant change compared to the mid-trimester value. SCA patients were mildly anemic with a mean Hb of 10.3 g/dl. In pregnant SCA patients, the mean Hb value was slightly lower in TM2 than in the non-pregnant SCA group but this was non-significant. There was a further slight reduction of the Hb level in TM3 in these patients, and this was significantly lower than the non-pregnant SCA subjects (*P < *0.007). The hemoglobin levels of both SCA patient groups were significantly lower than their corresponding normal counterparts (*P < *0.002).

Leucocyte counts were significantly higher in pregnant subjects and SCA patients compared to the healthy non-pregnant group. In both normal as well as SCA pregnancies, leucocyte counts increased in the third trimester and were significantly different from the respective control subjects and second trimester values (*P < *0.001). The highest leucocyte counts were seen in pregnant SCA subjects in the third trimester (mean 9.8 ± 3.7 × 10^9^/L). Hemoglobin fractions were identified by HPLC. The proportions of HbS and HbF were not significantly different between pregnant and non-pregnant SCA patients.

Coagulation studies showed minor, but significant differences between study groups (Table 1). In SCA patients, prothrombin time was slightly elevated, and the mean value was significantly higher compared to other groups (*P < *0.001). Although there were significant differences between the other groups, statistical comparison of the means showed no consistent pattern. In general, there was a tendency for shortening the PTT and TT during pregnancy. Pregnant SCA subjects showed a highly significant reduction of TT compared to healthy subjects and mid-trimester normal pregnancy (*P < *0.001).

**Table 1:** Results of coagulation studies in study subjects*

Study groups	PT (sec)	PTT (sec)	TT (sec)	Fibrinogen (mg/dl)	D-dimer (μg/l)
Healthy-NP	11 ± 0.75 (10–13)	28.9 ± 3.7 (24*–*38)	17.6 ± 1.6 (15.2–21.2)	282 ± 64 (184*–*423)	106 ± 77 (49*–*371)
Healthy-P					
TM2	10 ± 0.74 (10*–*12)	27.0 ± 2.0 (22*–*32)	16 ± 2.4 (13.5–24.4)	384 ± 82 (260*–*576)	112 ± 89 (49*–*389)
TM3	11.5 ± 0.97 (10–14)	27.7 ± 4.4 (21–44.2)	16.2 ± 3.7 (12.8–28.6)	447 ± 109 (308*–*699)	158 ± 119 (49*–*511
SCA	12.9 ± 1.0 (11.9*–*15)	27.9 ± 3.5 (23*–*35)	17.11 ± 4.9 (12–31)	263 ± 81 (157*–*444)	164 ± 104 (49*–*403)
SCA-P					
TM2	12.5 ± 0.71 (10.7*–*13)	28.1 ± 3.2 (22*–*35)	14 ± 0.9 (12.3–15.8)	350.1 ± 65 (247*–*479)	250 ± 190 (50*–*793)
TM3	11.5 ± 0.88 (9.8*–*14)	26.6 ± 3.5 (20.8*–*35)	14.4 ± 1.8 (11.4*–*19)	451 ± 149 (275*–*800)	427 ± 275 (58*–*1225)

*PT* prothrombin time, *PTT* partial thromboplastin time, *TT* thrombin time, *NP* non-pregnant, *P* pregnant, *SCA* sickle cell anemia, *Healthy-NP* healthy non-pregnant, *Healthy-P* healthy pregnant, *SCA-P* sickle cell anemia with pregnancy, *TM2* second trimester, *TM3* third trimester

*Values indicate mean ± SD (range)

Fibrinogen was consistently elevated in the pregnant subjects compared to the non-pregnant group. In normal pregnancy, the mean fibrinogen level was increased in TM2 and increased further in TM3 (*P < *0.001 compared to the healthy non-pregnant group). The fibrinogen level in SCA patients was not significantly different from the healthy non-pregnant subjects. With pregnancy in SCA, fibrinogen increased to significantly higher levels in TM2 and rose further in TM3 (*P < *0.001 versus both non-pregnant groups). Therefore, both in normal pregnancy and pregnancy associated with SCA, consistent elevation of fibrinogen was observed with pregnancy progression. However, there was no difference between non-pregnant SCA patients and their healthy counterparts.

In non-pregnant SCA patients, D-dimer levels were higher than those of the healthy non-pregnant subjects, with borderline significance (*P = *0.53). D-dimer was elevated in pregnancy. This was most significantly seen in SCA subjects with the progression of pregnancy. Consequently, D-dimer elevation was highly significant in the third trimester compared to the second trimester values of pregnant SCA subjects as well as the other two healthy groups (*P < *0.001 versus each group). The paired sample *t* test was used to compare coagulation parameters between the second and third trimesters of pregnancy. For healthy pregnant subjects, there was a significant increase from the second to third trimester in PT (*P < *0.01), fibrinogen (*P = *0.02), and D-dimer values (*P = *0.01). There was a marked increase from second to third trimesters of fibrinogen and D-dimer levels in the pregnant SCA group (*P < *0.01).

**Fig. 1:**
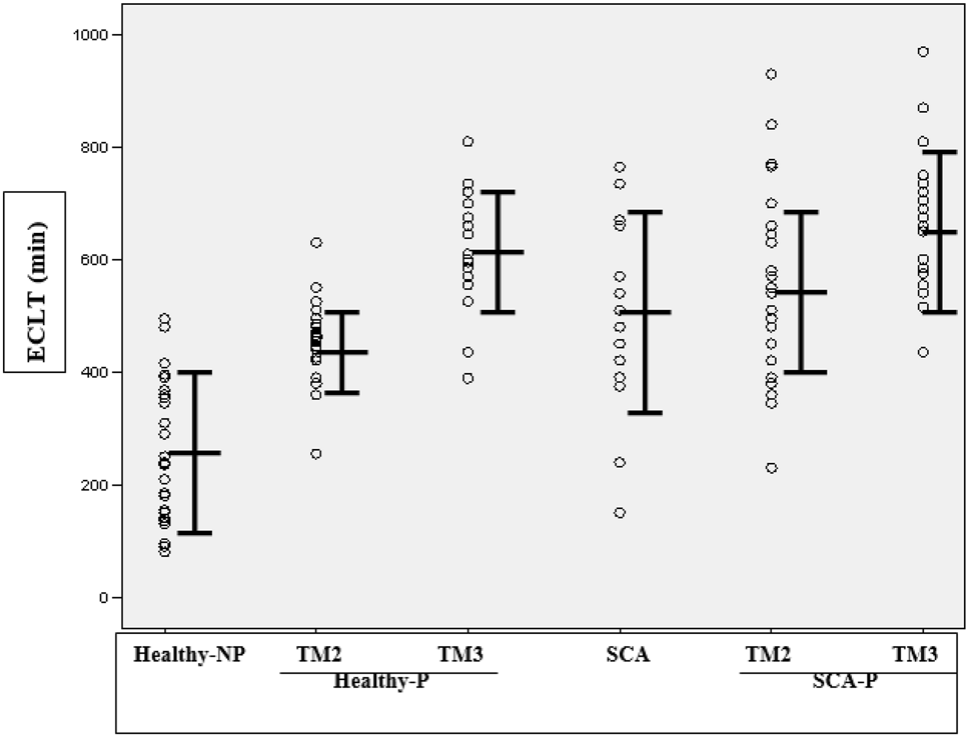
ECLT in study groups. *Healthy-NP* healthy non-pregnant, *Healthy-P* healthy pregnant, *TM2* second trimester; *TM3* third trimester; *SCA* sickle cell anemia, *SCA-P* sickle cell anemia with pregnancy. Bars indicate ranges corresponding to mean ± SD

Figure 1 shows the distribution of ECLT values in the different study groups. The mean ECLT in healthy non-pregnant subjects was 272 ± 125 min (range, 80*–*495 min). Clot lysis times observed in the study groups were significantly different from each other. Mean ECLT in TM2 was prolonged to 437 min in normal pregnancy and was further prolonged to 625 min in TM3. These differences were highly significant (*P < *0.001).

In the SCA group, mean ECLT (511 ± 160 min) was significantly higher than that of the healthy non-pregnant group (*P < *0.001). In pregnant SCA subjects, there was progressive elevation of ECLT over time with a significant difference between TM2 (548 min) and TM3 (666 min) values (*P < *0.001). ECLT in TM3 was significantly higher than the non-pregnant SCA subjects (*P < *0.001). However, ECLT in the third trimester of the two pregnancy groups were not significantly different from each other.

**Fig. 2:**
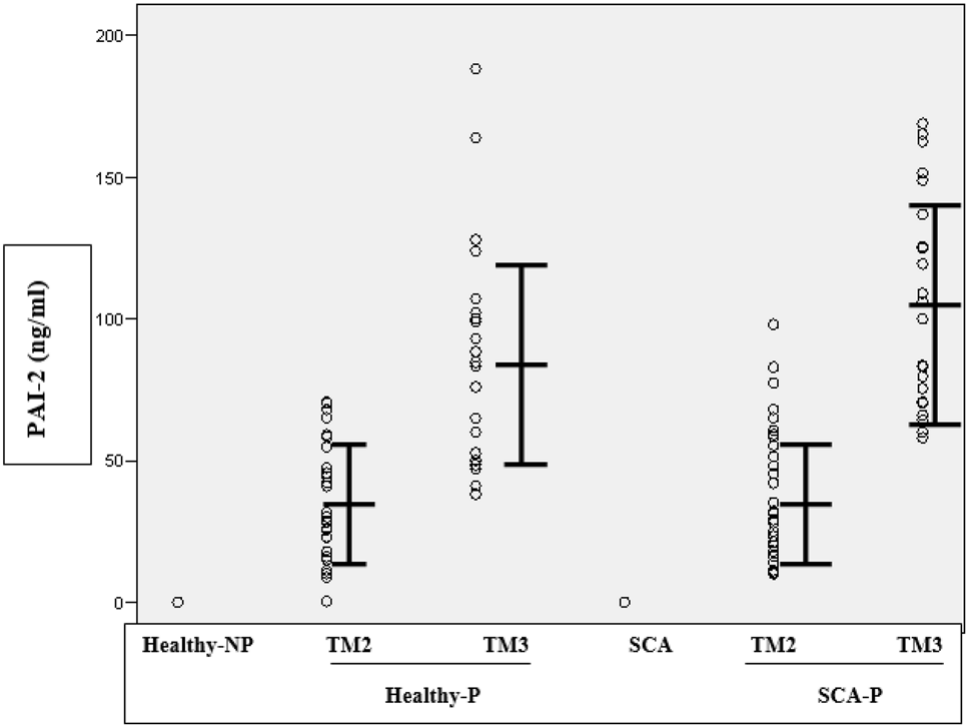
The distribution of PAI-2 levels in the study groups. *Healthy-NP* healthy non-pregnant, *Healthy-P* healthy pregnant, *TM2* second trimester, *TM3* third trimester, *SCA* sickle cell anemia, *SCA-P* sickle cell anemia with pregnancy. Bars indicate range corresponding to mean ± SD

Figure 2 shows the distribution of PAI-2 levels. PAI-2 level was below the detection limit in the non-pregnant groups, but it was detected with pregnancy in both healthy and SCA cases. There was a marked increase from TM2 to TM3 in both groups. These differences were all highly significant (*P < *0.001 respectively, paired samples *t* test). However, PAI-2 levels in each trimester in the healthy group were not significantly different as compared to corresponding trimesters in the SCA group although the mean PAI-2 level in the third trimester of SCA patients was higher than the healthy pregnant group (independent samples *t* test).

**Fig. 3:**
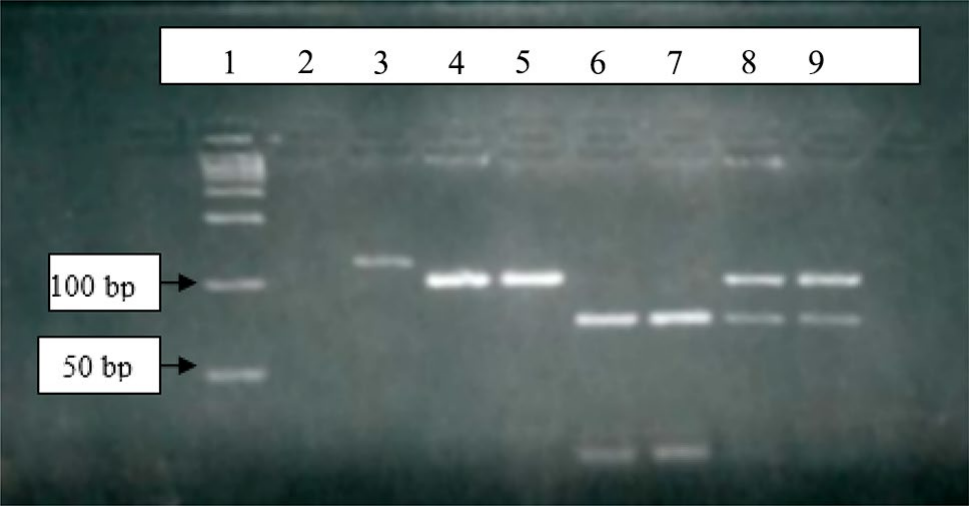
Determination of PAI-2 genotype by MwoI digestion of PCR product and electrophoresis on a 5% metaphore gel. Lane 1: 50 bp DNA ladder; lane 2: negative control; lane 3: PCR product; lane 4 and 5: AA homozygotes with digestion products of 108 bp; lane 6 and 7: BB homozygotes with bands of 78 bp and 30 bp; lane 8 and 9: AB heterozygotes with bands of 108 bp, 78 bp and 30 bp. The 15 bp digestion fragment was not visualized

Figure 3 shows MwoI digestion products that were obtained. The G to C substitution at position 413 in variant B created a new MwoI restriction site absent in variant A. A second MwoI restriction site was also present in the amplified region in both variants and resulted in a fragment of constant size (15 bp). The 123 bp PCR product was split into two fragments of 108 bp and 15 bp in AA homozygotes and three fragments of 78, 30, and 15 bp in BB homozygotes. Heterozygotes had all four bands, i.e., 108, 78, 30, and 15.

Genotype and allele frequencies observed in the total study population are shown in Tables 2 and 3. Homozygosity for the A allele showed the highest frequency (48.6%), while the homozygous B genotype was the least frequent (8.8%).

**Table 2:** Distribution of PAI-2 genotypes in study groups

PAI-2 genotype	Healthy-NP	Healthy-P	SCA	SCA-P	Total	χ^2^	*P *value*
AA	19 (61.3%)	16 (51.6%)	9 (45%)	11 (35.5%)	55 (48.6%)	5.17	0.52
AB	10 (32.2%)	13 (41.9%)	10 (50%)	16 (51.6%)	49 (43.3%)		
BB	2 (6.5%)	2 (6.5%)	1 (5%)	4 (12.9%)	9 (8.0%)		

*Healthy-NP* healthy non-pregnant, *Healthy-P* healthy pregnant, *SCA *sickle cell anemia, *SCA-P* sickle cell anemia with pregnancy.

*Chi-square test; significance at α < 0.05

**Table 3:** Distribution of allele frequencies in the study and control groups

PAI-2 allele frequency	Healthy-NP	Healthy-P	SCA	SCA-P	Total allele	χ^2^	*P *value**
A allele	0.774 (48)	0.726 (45)	0.700 (28)	0.613 (38)	(0.703) 159	4.08	0.253
B allele	0.226 (14)	0.274 (17)	0.300(12)	0.387 (24)	(0.296) 67		

*Healthy-NP* healthy non-pregnant, *Healthy-P* healthy pregnant, *SCA* sickle cell anemia, *SCA-P* sickle cell anemia with pregnancy

*Figures indicate allele frequencies (number of chromosomes); ***Chi-square test; significance at α < 0.05

Based on the observed allele frequency and assuming that the Hardy–Weinberg equilibrium is met, the expected genotype frequencies are shown in Table 4. Significant differences between the observed and expected values were assessed by an exact test using a Markov chain provided by the Arlequin program. No significant differences from Hardy–Weinberg equilibrium were observed. The distributions of the PAI-2 genotypes within the study groups were not significantly different (χ^2^ = 5.17, *P = *0.52).

**Table 4:** PAI-2 genotypes in study subjects: assessment of Hardy–Weinberg equilibrium

Study groups	Values	AA	AB	BB	χ^2^	*P* value*
Healthy-NP						
	Observed	19	10	2	1.08	0.582
	Expected	15.1	13.4	2.5		
Healthy-P						
	Observed	16	13	2	0.16	0.923
	Expected	15.1	13.4	2.5		
SCA						
	Observed	9	10	1	0.54	0.763
	Expected	9.7	8.7	1.6		
SCA-P						
	Observed	11	16	4	1.3	0.522
	Expected	15.1	13.4	2.5		

*NP* non-pregnant, *P* pregnant, *SCA* sickle cell anemia, *Healthy-P* healthy pregnant, *SCA-P* sickle cell anemia with pregnancy

*Exact test using a Markov chain provided by the Arlequin program; significance at α < 0.05

Table 5 shows PAI-2 levels in relation to the polymorphism type in both pregnant groups (normal and SCA). The mean values of PAI-2 levels in the individual polymorphic groups AA, AB, and BB increased from the second to the third trimester. This was consistent in both pregnancy groups. One-way ANOVA showed no significant association between PAI-2 genotypes and PAI-2 levels in both groups.

**Table 5:** Distribution of plasma PAI-2 antigen levels (ng/ml) in the PAI-2 Ser (413)/Cys polymorphic groups

Study groups	AA	AB	BB
Healthy-P (n = 25)			
TM2	38.27 ± 27.04	40.32 ± 17.54	57.0 ± 2.83
TM3	85.05 ± 23.62	80.83 ± 48.89	164 ± 0
SCA-P (n = 30)			
TM2	45.02 ± 26.9	39.19 ± 21.02	21.6 ± 5.2
TM3	103.51 ± 44.22	100.35 ± 33.93	92.07 ± 51.95

*P* pregnant, *TM2* second trimester, *TM3* third trimester, *Healthy-P* healthy pregnant, *SCA-P* sickle cell anemia with pregnancy

*Values indicate mean ± SD

Maternal and fetal complications were documented in all pregnancies followed up to delivery at SMC. The outcome measures were episodes of crisis requiring hospital admission, antenatal and postnatal pregnancy complications, and fetal and neonatal complications (Table 6). The maternal complications included in the analysis were preeclampsia, eclampsia, anemia, premature rupture of the membrane, preterm delivery, and maternal mortality. Fetal and neonatal complications included low birth weight, stillbirth, intrauterine growth retardation, and neonatal death.

**Table 6:** Pregnancy-related outcomes in healthy subjects and sickle cell anemia patients

Study groups	Gestation period (weeks)	Preeclampsia	HTN	Acute anemic event	Premature membrane rupture	Premature labor	Rupture of the uterus	Neonatal death	Low birth weight	Total complications
Healthy-P (n = 25)	38.3 ± 1.5 (34–40)	1	3	1	0	2	0	0	3	10
SCA-P (n = 30)	37.1 ± 2.3 (29–40)	0	0	7	1	2	1	1	3	15

HTN, pregnancy-related hypertension,* Healthy-P* healthy pregnant, *SCA-P *sickle cell anemia with pregnancy

### Period of gestation

Comparison between periods of gestation in the two pregnancy groups showed a lower average gestation period in the SCA group (37.1 ± 2.3) than normal pregnant subjects (38.3 ± 1.5), although not statistically significant.

### Episodes of sickle crisis

There were only three episodes of sickle cell crisis in the test group requiring hospital admission. This was calculated as 2.6 episodes per 1000 patient weeks. A review of computerized hospitalization records showed a total of six crisis admissions for this group during the 52 weeks *prior* to the last menstrual period (LMP) for these cases (3.8 episodes for a thousand weeks). The difference was not statistically significant suggesting that pregnancy did not increase the risk of sickle crisis in these patients.

### Transfusion episodes

In the group of pregnant SCA patients, the total number of hospitalizations for transfusions was 46 during pregnancy, whereas the same patients required 8 during the year preceding pregnancy. The corresponding calculated averages were 41.3 and 5.1 transfusion episodes per 1000 patient weeks. The difference was highly significant (*P < *0.0001).

The distribution of maternal and fetal complications in the healthy group refers to 25 out of 31 normal pregnancies that were delivered at the SMC. Seven maternal complications included one case of preeclampsia, three of pregnancy-induced hypertension, one of anemia, and two cases of premature labor. The pregnancy outcome in this group included three low birth weight neonates. No neonatal death was observed. In comparison, there were eleven complications in the pregnant SCA group that included seven acute anemic events, one premature rupture of the membrane, two premature labors and one uterine rupture. Neonatal complications included three low birth weight infants and one intrauterine fetal death.

Appearance, Pulse, Grimace, Activity, and Respiration Scores (APGAR scores) of all neonates at 1 min and 5 min after delivery were assessed in all cases (healthy group: 1st min 8.8 ± 0.63, 5th min 9.9 ± 0.2; and SCA: 1st min 8.9 ± 0.37, 5th min 9.9 ± 0.37). Maternal and fetal complications and APGAR scores were not significantly different between the two groups. There was no association between maternal and fetal complications with PAI-2 levels in both pregnancy groups.

## Discussion

Results from this study indicate that SCA in Bahrain is hematologically similar to that in eastern Saudi Arabia, with similar Hb levels, whereas the disease is generally more severe in the western region of Saudi Arabia [1]. Coagulation tests in pregnant normal and pregnant SCA patients, revealed a mild shortening of PTT and TT in some cases which indicates the presence of a hypercoagulable state in these patients. However, results from the second to third trimester differed between the groups. These observations are consistent with earlier studies and are likely the result of increased coagulation factors VII, VIII, X, XII, XIII, VWF, and fibrinogen that is attributed to increased estrogen levels in pregnancy [12].

Fibrinogen levels increased in pregnancy, and there was a significant difference between healthy pregnant and healthy non-pregnant subjects, which was consistent with existing data [12, 17, 18]. Similar to previous research, the mean fibrinogen level in the SCA group was not significantly different from the healthy non-pregnant groups [19, 20]. In the pregnant SCA subjects, fibrinogen was significantly elevated in the second trimester and increased further in the third trimester. The levels were not significantly different as compared to the healthy pregnant group. High fibrinogen levels combined with activated clotting factors (suggested by reduced TT) provide a setting of increased coagulability in these patients. Though highly significant compared to normal non-pregnant subjects, the differences were non-significant compared to the situation in normal pregnancy. There is no available data on fibrinogen levels in SCA associated with pregnancy for comparison in the literature.

D-dimers are specific plasmin degradation products of fibrin, and elevated levels above normal are seen in conditions such as deep vein thrombosis with or without thromboembolism [20, 21]. Normal pregnancy causes the maternal plasma D-dimer concentration to increase progressively from conception until delivery [18]. However, the literature lacks evidence for a diagnostic D-dimer concentration threshold to discriminate pregnant women at high risk of venous thromboembolism [18]. Findings in the current study showed that elevated baseline D-dimer in steady-state SCA was significantly elevated in the second trimester in the pregnant SCA subjects with further increase in the third trimester. These values were significantly different from other groups, suggesting a higher level of thrombin activation, fibrin generation, and consequent fibrinolysis in these patients, similar to the observations in SCA patients in crisis in other studies. Clinically, none of these patients had a crisis at the time of sampling. Furthermore, frequency of crises was also not increased during pregnancy. Therefore, it is likely that other balancing factors were at work in pregnant SCA patients preventing the pathological consequences of the hyperactive coagulation system. No comparable studies were found in the literature.

Lysis time of the euglobulin clot was used to estimate the total fibrinolytic activity in plasma samples. A positive test is considered to be premature disappearance of the clot (usually within 60–120 min), suggesting increased fibrinolysis, although the specific cause of excessive fibrinolysis is not determined using this technique [22]. We found a marked reduction of fibrinolytic activity and prolongation of ECLT in normal pregnancy with a highly significant difference between the healthy pregnant and healthy non-pregnant groups. ECLT mainly reflects the interaction between t-PA and PAI-1, while t-PA activity decreases during pregnancy due to an increase in PAI-1 and PAI-2 [16, 23]. This partially explains the prolongation of ECLT in pregnant women. Steady-state SCA patients showed significantly longer ECLT compared to the normal group, indicating inhibition of fibrinolytic activity in these patients. This observation is common in other studies [24, 25]. In the pregnant SCA group, ECLT was highly prolonged in the second trimester, and it was significantly higher than in the healthy pregnant group. However, this difference between the two pregnant groups in the third trimester was no more significant due to a comparatively higher third trimester increase in the normal subjects. There is no available data on ECLT changes in SCA associated with pregnancy for comparison in the literature.

The results of PAI-2 assay in this study are consistent with previous studies that have shown a marked temporal increase of PAI-2 levels in normal pregnancy [23, 26, 27]. The increase in plasma PAI-2 levels during pregnancy progression is explained by its placental origin with levels increasing in parallel with the growth of the placenta. Both SCA pregnancy and preeclampsia are associated with placental infarction and insufficiency leading to intrauterine growth retardation [28]. Significant reductions in antigenic and functional PAI-2 have been reported in severe preeclampsia compared to normal pregnancies in the third trimester. In addition, PAI-2 levels were reduced in patients with preeclampsia and placental infarction compared to patients without infarction [28]. Infarction would lead to more severe placental insufficiency and reduce the production of PAI-2 further. In this study, 2 out of 6 pregnancies with low birth weight infants had low levels of PAI-2 in the second trimester, indicating a possible role of placental insufficiency in these cases. However, it is important to note that in our pregnant SCA patients, no such reduction was observed. Indeed, mean PAI-2 was higher in this group (though not statistically significant) as compared to the healthy pregnancy group suggesting that most patients likely had no or insignificant placental insufficiency or infarction. Moreover, gross infarction during pathological examination of the placenta was not detected at delivery. On the other hand, it may be argued that whereas reduction of PAI-2 may be a consequence of placental infarction, impaired fibrinolysis due to elevated PAI-2 could be a risk factor for vascular complications.

Similar to observations in normal pregnancy, mean PAI-2 levels in pregnant SCA patients showed a marked increase from the second (38.9 ng/mL) to the third trimester (100.4 ng/mL). Although the results of coagulation testing, PAI-2 assay and increased ECLT suggest a high risk of thromboembolic complications in SCA associated with pregnancy, in our patients this did not translate into significantly higher frequency of feto-maternal complications or any evidence of placental insufficiency. Therefore, it may be hypothesized that counterbalancing factors operate to reduce this risk. Coagulation testing only reflects plasma levels of factors, but not the situation at the level of the endothelial surface in placental blood vessels. For example, one such mechanism could be compensatory u*P-*regulation of thrombomodulin and protein C receptor on the surface of endothelial cells observed in a sickle cell mouse model [29]. There are no comparative studies in the literature on PAI-2 levels in SCA pregnancy.

There is relatively little data on the relevance of PAI-2 polymorphism in clinical disorders. The distribution of PAI-2 genotypes in the healthy non-pregnant control group in this study (AA 61.3%, AB 32.3% and BB 6.5%) was not significantly different from the genotype distribution in persons of European descent (AA 64%, AB 32.2% and BB 3.8%) as observed by Foy et al. (1997). The distribution of PAI-2 allele frequencies in the healthy non-pregnant control group was similar to that of European volunteers in the study by Foy et al. (1997) [3].

The percentage of AA genotype in this study was greater than that found by Mercado et al. (2007) in the control group of Mexican origin (AA:34%) [11]. The frequency of the B allele in Pima Indians was significantly higher than the Europeans in the latter study. The frequency of the A allele also appears to be greater than observed in Mexicans (A 0.57 and B 0.43) [11] and normal Turkish controls in the study by Buyru et al. (2003) [10]. The current study shows no association between the PAI-2 genotypes and PAI-2 levels as observed in the pregnant subjects, both SCA and healthy controls. Larger studies would be required to validate these observations. This is the first study that evaluated PAI-2 genotypes in pregnant SCA patients. Therefore, there is no available data from the literature to compare with the results of this study.

Pregnancy in patients with SCA is associated with an increased risk of medical complications during pregnancy. The maternal risks include prepartum and postpartum painful crises, urinary tract infections, pulmonary complications, anemia, preeclampsia and death. Fetal complications include premature delivery with its associated risks, intrauterine growth retardation (low birth weight), fetal distress during labor and a high perinatal mortality rate [30–33]. No significant differences in maternal, fetal or neonatal complications were observed in the present study between SCA pregnancy and normal pregnancy. The average number of sickle cell crisis episodes experienced by the group during pregnancy was also lower, although not significantly, compared to the previous year. The only significant increase in pregnancy-related morbidity was anemia requiring transfusion support. In this regard, several important remarks should be made: (a) this study included patients in the second trimester; spontaneous abortions were not included in the list of complications. Thus, second trimester pregnancies may exclude more severe SCD cases, leading to bias. (b) Many relatively recent studies show no maternal mortality. The high mortality rate in Africa may be primarily due to limited health care and prenatal care resources [33].

## Conclusion

PAI-2 antigen levels increased more steeply from the second to third trimester in pregnant Bahraini SCA patients compared to their normal counterparts although mean levels were not significantly different. Moreover, significant coagulation system activation with ongoing fibrinolysis in a background of impaired fibrinolysis characterized pregnant SCA patients. No correlation was observed between PAI-2 genotypes and plasma antigen levels. Despite the heightened hypercoagulability risk in pregnant Bahraini SCA patients, the outcome of their pregnancy appears to be considerably better than that reported in other populations such as Nigeria and Benin and suggests a possible role of compensatory anticoagulant mechanisms.

## Limitations and future research

As this was the first study to determine antigen levels in plasma and genotypes of PAI-2 in pregnant and non-pregnant homozygous SCA patients, it should be considered in light of the limitations. The first limitation is related to the small sample size. Therefore, the observations and results presented in this article should prompt further study of blood coagulation and fibrinolysis in pregnant patients with SCA. Moreover, if a dramatic increase in PAI-2 and impaired fibrinolysis in the second trimester in patients with SCA is correlated with placental pathology and malperfusion, the possible role of therapeutic use of plasminogen activators or drug-targeting PAI-2 in high-risk patients can be explored. Findings of this study assume greater significance in view of evidence that the incidence of venous thromboembolism in pregnant sickle patients is significantly higher than in pregnancy-related VTE in the general population [34]. Hence, a multi-institutional and multi-ethnic study would have sufficient strength to clarify some of the issues raised in this study and eliminate apparent differences.

## Data Availability

The data that support the findings of this study are available on request.
